# Effects of glucosamine in patients with osteoarthritis of the knee: a systematic review and meta-analysis

**DOI:** 10.1007/s10067-018-4106-2

**Published:** 2018-04-30

**Authors:** Toru Ogata, Yuki Ideno, Masami Akai, Atsushi Seichi, Hiroshi Hagino, Tsutomu Iwaya, Toru Doi, Keiko Yamada, Ai-Zhen Chen, Yingzi Li, Kunihiko Hayashi

**Affiliations:** 1 National Rehabilitation Center for Persons with Disabilities, 4-1, Namiki, Tokorozawa, Saitama Japan; 2 Gunma University Initiative for Advanced Research, 39-15, Showa-cho, Maebashi, Gunma Japan; 3 International University of Health and Welfare, 1-3-3, Minamiaoyama, Minato-ku, Tokyo, Japan; 4 Department of Orthopaedic Surgery, Mitsui Memorial Hospital, 1, Kandaizumi-cho, Chiyoda-ku, Tokyo, Japan; 5 Rehabilitation Division, Tottori University Hospital, 36-1, Nishimachi, Yonagoshi, Tottori Japan; 6 School of Health Science, Faculty of Medicine, Tottori University, 36-1, Nishimachi, Yonagoshi, Tottori Japan; 7 Nagano University of Health & Medicine, 11-1, Imaihara, Kawanakajimacho, Naganoshi, Nagano Japan; 8 Department of Orthopaedic Surgery, Graduate School of Medicine, The University of Tokyo, 7-3-1, Hongo, Bunkyo-ku, Tokyo, Japan; 9 School of Health Sciences, Gunma University, 39-15, Showa-cho, Maebashi, Gunma Japan; 10 NPO International Eco-Health Research Group, 133-4, Horiguchicho, Isesakishi, Gunma Japan

**Keywords:** Glucosamine, Knee OA, Osteoarthritis, WOMAC

## Abstract

Osteoarthritis (OA) of the knee is one of the main causes of mobility decline in the elderly. Non-surgical treatments such as administration of supplements to strengthen the joint cartilage matrix have become popular not only for pain relief but also for joint preservation. Glucosamine has been used in many countries based on the increasing evidence of its effectiveness for OA. Although there are many previous studies and systematic reviews, the findings vary and different conclusions have been drawn. We aimed to review recent randomized controlled trials on glucosamine for knee OA to reveal up-to-date findings about this supplement. We also performed a meta-analysis of some of the outcomes to overcome the unsolved bias in each study. Eighteen articles written between 2003 and 2016 were analyzed. Many used visual analogue scale (VAS) pain scores and the Western Ontario and McMaster Universities Osteoarthritis Index (WOMAC), which were assessed in our meta-analysis. We found a marginally favorable effect of glucosamine on VAS pain scores. The effect on knee function, as measured by the WOMAC, was small and not significant. A newly established knee OA scale, the Japanese Knee Osteoarthritis Measure (JKOM), is commonly used in Japan. Although the number of subjects was small, the JKOM meta-analysis indicated that glucosamine is superior to a placebo in alleviating knee OA symptoms. Given this, we concluded that glucosamine has the potential to alleviate knee OA pain. Further studies are needed to evaluate the effect of glucosamine on knee function and joint preservation, as well as to evaluate the combined effect with other components, such as chondroitin.

## Introduction

Osteoarthritis (OA) is one of the main health problems in middle-aged and elderly populations because of its high prevalence and effect on activities of daily living. The pathological changes start in the cartilage of the joints, particularly in the weight-bearing joints such as the knees, hips, and vertebra. Recent surveys have reported that the prevalence of symptomatic knee OA is 12% in Americans older than 25 [1] and that radiographic changes (worse than Kellgren-Lawrence Grade 2) in knee joints are seen in 42.6% of men and 62.4% of women aged older than 40 in Japan [2]. The common symptoms of knee OA, such as pain, joint contracture, malalignment, and muscle weakness, lead to declined mobility. Yoshimura reported that people with symptomatic knee OA have a significantly lower physical quality of life (QOL) than those without it [3]. With the global aging population, the medical cost of these disorders has a large effect on health policies in each country.

Treatment modalities for knee OA have been established, implemented, and are documented in several guidelines [4–6]. The treatment modalities are largely divided into surgical and non-surgical treatments. Surgical knee joint arthroplasty is performed for more than 700,000 patients in the USA annually [7]. Non-surgical treatments include intra-articular injection, oral medication, plasters, exercise, and oral supplements. As an oral medication, non-steroidal anti-inflammatory drugs have been proven effective and are widely prescribed. Although these medical treatments have proven effectiveness, other therapeutic options have been proposed to use biological compounds, such as hyaluronans, chondroitin sulfate, and glucosamine, as oral supplements. The possibility that these compounds may have a chondroprotective effect on knee OA attracts significant interest among patients with knee OA.

Glucosamine is a biological component of joint cartilage and has been recognized in the USA, Europe, and Asian countries, as a supplement for knee OA together with chondroitin sulfate [8]. Numerous trials, as well as systematic reviews, related to glucosamine have been performed, and their conclusions are varied [8–14]. Two important reviews, Cochrane review in 2005 and the review by Eriksen et al. in 2014, reported the importance of the brand of glucosamine to explain the variance and that the studies using the Rottapharm/Madaus product showed statistically significant effects on knee OA symptoms, while other glucosamine products failed to prove their effects [8, 14]. The finding was reflected in the consensus statement of the European Society for Clinical and Economic Aspects of Osteoporosis and Osteoarthritis in 2014, which recommends the use of the prescription formulation of patented crystalline glucosamine sulfate (Rottapharm/Madaus product) and chondroitin sulfate [15]. However, besides the Rottapharm/Madaus glucosamine product, new products containing glucosamine have been emerging every year globally. Therefore, it is still of interest for many people to obtain up-to-date evidence for the effect of glucosamine in general. Moreover, as for the coverage of past reviews, search protocols were limited to articles written in English, and they did not include studies reported in Asian languages. Considering the popularity of glucosamine in Asian countries, studies from Asia are expected to provide additional information on the effectiveness of glucosamine.

In this study, we performed a systematic review of recent RCTs and other studies of glucosamine written not only in English but also in Japanese and Chinese, with a view of updating the current body of evidence on the effectiveness of glucosamine. We believe that the integration of independent RCTs in our meta-analysis will reveal non-biased outcomes regarding various glucosamine products and provide critical information for potential users of glucosamine with OA.

## Method

### Data sources

This study was performed based on a predefined and registered protocol (PROSPERO 2016: CRD42016036998). The authors searched for articles published as original studies, which appeared to provide useful information with regard to our research question, “In patients with knee osteoarthritis, what are the effects of glucosamine on pain and function?”

### Study Selection and Search Strategy

We searched electronic databases (Medline, Embase, Cochrane library, Cumulative Index to Nursing and Allied Health Literature, and Japan Medical Abstracts Society Database) for articles written in English, Japanese, and Chinese between 2003 and 2016. We also manually searched the references of relevant studies.

First, a reviewer assessed whether each article met our criteria, that is, an RCT on the effects of glucosamine on knee OA. The search keywords included “RCT,” “knee,” “osteoarthritis,” “glucosamine,” and their synonyms. We included a published RCT paper in the first screening so long as it contained a treatment arm of glucosamine administration. Second, the collected articles were reviewed by two experts in bone and joint surgery. The articles were examined thoroughly to extract information about study design, outcomes, and obtained data. The quality of each study was also examined. Quality and risk of bias were assessed using Cochrane’s risk of bias tool (sequence generation, allocation concealment, blinding of participants, personnel and outcome assessor, intention to treat, incomplete outcome data, selective outcome reporting, early cessation of the study, and other potential sources of bias).

### Statistical analyses

Standardized mean difference effect sizes were obtained by dividing changes from baseline (or differences between before and after treatment) by the pooled standard deviation of the visual analog scale (VAS) pain scores, Western Ontario and McMaster Universities Osteoarthritis Index (WOMAC) scores, and Japanese Knee Osteoarthritis Measure (JKOM) scores. Results for the comparative effect between the glucosamine group and control group were presented by standardized mean difference (SMD) estimates and 95% confidence intervals (95% CI). We used a standard inverse variance random effects model for meta-analysis. Publication biases and small study effects were assessed using conventional funnel plots. Heterogeneity was assessed by *I*^2^ statistics. A two-sided *p* value of < 0.05 was considered statistically significant. All statistical analyses were performed using Cochrane Review Manager software (RevMan) ver5.1 (The Nordic Cochrane Center, The Cochrane Collaboration) and SAS version 9.3 (SAS Institute, Cary, NC, USA).

## Results

### Selection process of articles

**Fig. 1:**
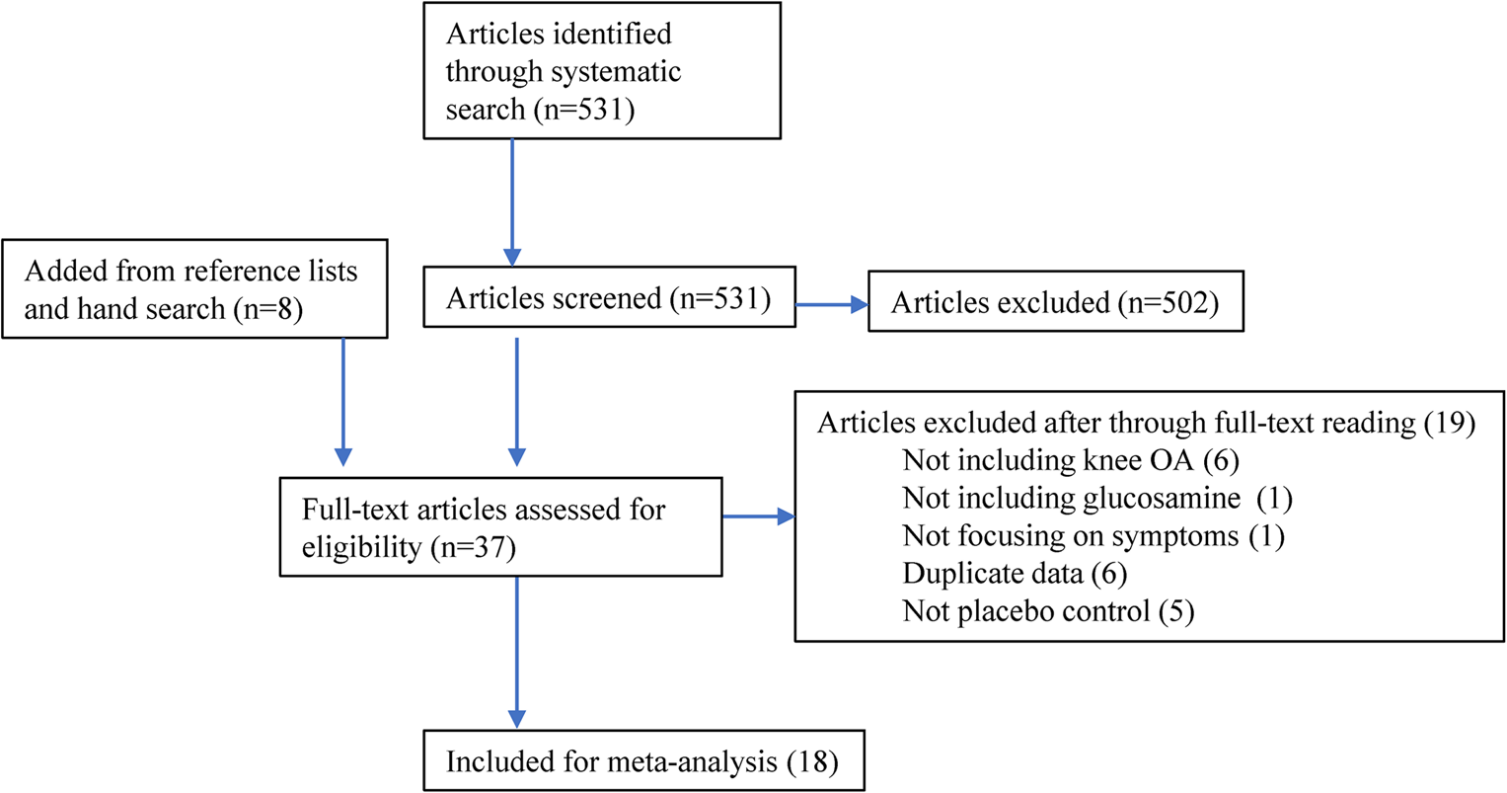
Study flow diagram

First, we identified 531 articles from the MeSH database that were related to the effect of oral supplements for OA. In addition, we found four systematic reviews [8, 12–14]. After title and abstract review, we obtained 29 articles related to glucosamine intervention. Further, we included additional 8 articles identified via manual search and obtained 37 articles for the second screening. After a thorough full-text reading process by experts, 19 articles were removed and 18 articles were used for further analyses (Fig. 1) [16–33]. Table 1 shows the list of obtained RCT articles. While nine studies used glucosamine alone, nine studies used commercial supplements containing both glucosamine and other supplements such as chondroitin (included in six studies). The formula of glucosamine is also varied; five studies used sulfate salt of glucosamine (two of them used Rottapharm/Madacus-made crystalline of sulfate salt), six studies used hydrochloride salt, and one study used *N*-acetylated glucosamine (unclear for the rest of the studies). Among 18 studies, 9 studies were performed in Japan (6 articles were written in Japanese). We found one Chinese RCT, but the study compared different dosages of glucosamine and was excluded from the analysis [34].

**Table 1:** Demographic characteristics of trials

Author [ref]	Country	Participants (*n*, drop out)	Intervention	Control	Outcome	Study duration (weeks)	Allocation concealment	Blinding
Hayami et al. [16]	Japan	20, 10%	Glucosamine containing supplement^a^	Placebo	JOA knee score	8	Unclear	Adequate
Usha et al. [17]	India	58, 12%	Glucosamine	Placebo	VAS, Lequesne index	12	Unclear	Adequate
Cibere et al. [18]	Canada	137, 2%	Glucosamine	Placebo	WOMAC, EQ-5D	24	Adequate	Adequate
McAlindon et al. [19]	USA	205, 9%	Glucosamine	Placebo	WOMAC	12	Unclear	Adequate
Kajimoto et al. [20]	Japan	23, 0%	Glucosamine containing supplement^a^	Placebo	JOA knee score	12	Unclear	Adequate
Clegg et al. (GAIT) [21]	USA	630, 22%	Glucosamine	Placebo	WOMAC, OMERACT-OARSI, HAQ	24	Adequate	Adequate
Hatano et al. [22]	Japan	78, 14%	Glucosamine containing supplement	Placebo	VAS	12	Unclear	Adequate
Herreo-Beaumont et al. [23]	Spain and Portugal	210, 30%	Glucosamine	Placebo	WOMAC, Lequesne index, OARSI	24	Adequate	Adequate
Frestedt et al. [24]	USA	31, 32%	Glucosamine	Placebo	WOMAC, 6 MWD	12	Unclear	Adequate
Inuzuka et al. [25]	Japan	18, 11%	Glucosamine containing supplement	Placebo	VAS	6 (cross over)	Adequate	Adequate
Nakasone et al. [26]	Japan	32, 0%	Glucosamine containing supplement^a^	Placebo	VAS, JKOM	16	Unclear	Adequate
Kanzaki et al. [27]	Japan	40, 3%	Glucosamine containing supplement^a^	Placebo	VAS, JOA criteria	16	Adequate	Adequate
Gokan et al. [28]	Japan	18, 0%	Glucosamine	Placebo	VAS, JKOM	6	Unclear	Adequate
Nieman et al. [29]	USA	108, 6%	Glucosamine containing supplement	Placebo	WOMAC, VAS, SF-36, 6-MWD	8	Unclear	Adequate
Yokoi et al. [30]	Japan	34, 24%	Glucosamine	Placebo	VAS, JOA criteria	12	Unclear	Adequate
Fransen et al. (LEGS) [31]	Australia	303, 16%	Glucosamine	Placebo	WOMAC, VAS, SF-12, JSN	2 years	Adequate	Adequate
Tsuji et al. [32]	Japan	50, 44%	Glucosamine containing supplement^a^	Placebo	VAS, JKOM, 6-MWD	12	Adequate	Adequate
Sterzi et al. [33]	Italy	53, 6%	Glucosamine containing supplement^a^	Placebo	WOMAC, VAS, Lequesne index	12	Unclear	Adequate

^a^Chondroitin is involved in the supplement, as well as glucosamine

### Trial demographics of the selected articles

Among 18 identified RCTs, the study size varied between 18 and 630 subjects. We found 6 RCTs that included more than 100 subjects, while 8 RCTs involved less than 50 subjects. The duration of observation varied from 6 weeks up to 2 years, with 12 weeks being the most common observation period (8 studies). Only one study exceeded 1 year of observation and the study set joint preservation as its outcome [31]. Notably, we found several follow-up studies of studies performed previously [35–37]. Therefore, we only selected studies that were published for the first time after 2003, and follow-up studies were excluded.

As for the outcome measures, all studies included some sort of pain scale. The most commonly used outcome scale for health-related QOL was the WOMAC, which was used in eight RCTs. In the studies performed in Japan, another patient-oriented questionnaire, JKOM, was used in three RCTs. This scale is based on WOMAC and SF-36 and designed to evaluate pain, limitation in mobility related to daily activity, and restriction of participation as separate domains [38]. The Japanese Orthopaedic Surgery Association Knee rating score (JOA score), which is a forerunner to the JKOM, was also used in three RCTs in Japan. Note that the entire WOMAC was not always used in each study. Many studies utilized both the WOMAC total score and sub-scores including pain, stiffness, and physical function.

We assessed the risk of bias in included studies by reviewing the methodological quality of each study. While all the studies adequately described the randomization process and blinding, only seven studies described concealment of allocation.

### Effect of intervention and meta-analysis

Overall, 12 studies (67% of reviewed) concluded that glucosamine was effective compared with a placebo control. It is noteworthy that four of six RCTs with large number of subjects (*n* ≥ 100) concluded that glucosamine was not superior to a placebo.

### Effect of glucosamine on pain in knee OA

**Fig. 2:**
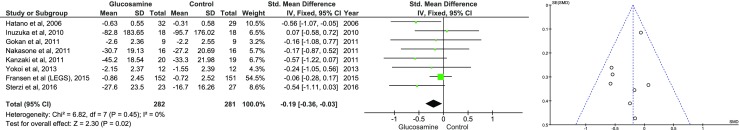
Changes in visual analog scale pain score in patients treated with glucosamine versus placebo. Funnel plot shows effect size versus standard error of effect size. Generally, the smaller the study, the bigger the standard error of effect size. Dotted lines represent the expected variation of effect size in comparison to standard error. SMD: standardized mean difference

**Fig. 3:**
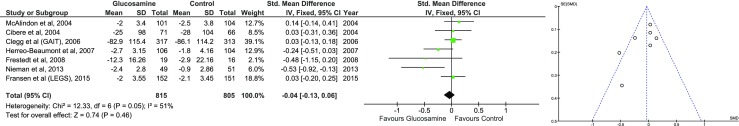
Changes in WOMAC pain sub-score in patients treated with glucosamine versus placebo

While all the reviewed papers included the assessment of the effect of glucosamine on pain, 10 studies used a VAS pain score, 7 used the WOMAC pain sub-score, and 3 used other scales. In one study [32], the baseline pain scores were significantly different between the glucosamine group and the control group. In another study, the baseline pain scores were not presented [17]. Therefore, we excluded these two studies from the primary analyses of pain scores. As shown in Fig. 2, the summarized analysis of VAS pain scores shows an effect size of − 0.19 (95% CI − 0.36, − 0.03), suggesting a statistically significant favorable effect of glucosamine. The funnel plot (Fig. 2) indicated relatively small inconsistency. As for WOMAC pain scores (Fig. 3), the summarized effect size was − 0.04 ([95% CI] − 0.13, 0.06), suggesting a favorable but not significant effect of glucosamine on knee pain as measured by WOMAC. In a previous systematic review, a large inconsistency had been reported, and the effect of different brands was considered an important factor [14]. In contrast to previous reviews in which numerous trials used the Rottapharm/Madaus product, which tended to provide better results than other products, we only included 2 of 18 trials which used this brand. Herreo-Beaumont reported favorable results for glucosamine from Rottapharm/Madaus [23], while McAindon reported non-significant effects of glucosamine partially supplied by the same company [19]. Neither of the summarized effect of two Rottapharm/Madaus product studies nor the rest of studies showed significant effect in WOMAC pain sub-score (data not shown).

**Fig. 4:**
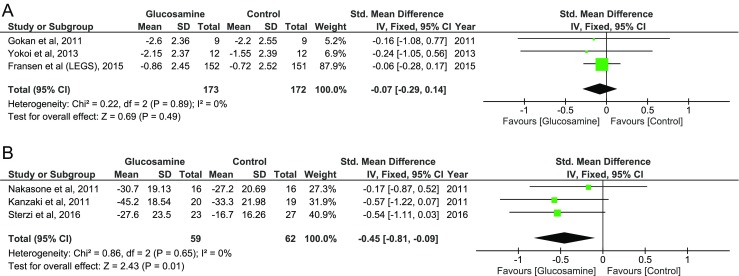
Changes in visual analog scale pain score in patients treated with glucosamine alone or glucosamine with chondroitin. **a** Summarized effect size of three studies which used glucosamine alone. **b** Summarized effect size of studies which used glucosamine and chondroitin

To examine the combined effect of glucosamine and chondroitin, we separated the studies into glucosamine alone and glucosamine with other components including chondroitin. As shown in Fig. 4, while three glucosamine alone studies showed non-significant effect on pain with effect size of − 0.07 (95% CI, − 0.29, 0.14), studies using glucosamine and chondroitin show an effect size of − 0.45 (95% CI, − 0.81, − 0.09), indicating significant effect on pain.

### Effect of glucosamine on health-related QOL among patients with knee OA

**Fig. 5:**
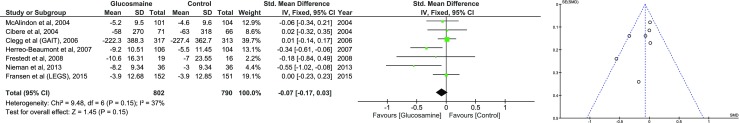
Changes in Western Ontario and McMaster Universities Osteoarthritis Index physical function sub-score in patients treated with glucosamine versus placebo

**Fig. 6:**
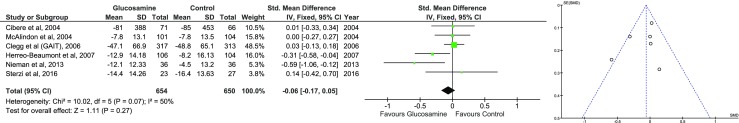
Changes in Western Ontario and McMaster Universities Osteoarthritis Index total score in patients treated with glucosamine versus placebo

The health-related QOL among patients with knee OA was assessed with several outcome scales, among which WOMAC and JKOM were used for our meta-analysis. As for WOMAC, the physical function sub-score and total score showed similar results (Figs. 5 and 6, respectively). The summarized effect size was − 0.07 ([95% CI] − 0.17, 0.03) for the physical function sub-score and − 0.06 ([95% CI] − 0.17, 0.05) for the total score. For both cases, studies by Herrero-Beaumont et al. and Nieman et al. showed relatively larger effect sizes than others did in funnel plots [23, 29]. The summarized data suggest that glucosamine has a small, non-significant effect size on health-related QOL measured by WOMAC.

**Fig. 7:**
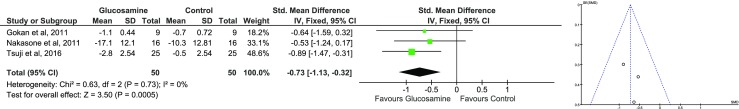
Changes in Japanese Knee Osteoarthritis Measure score in patients treated with glucosamine versus placebo

As for JKOM, another health-related QOL scale, the summarized effect size was − 0.73 ([95% CI] − 1.13, − 0.32), indicating favorable results for glucosamine (Fig. 7). All studies using the JKOM took place in Japan, and one of the three trials was supported by the company that provided the glucosamine product. None of the three trials used glucosamine alone but used it in combination with other supplements, including chondroitin sulfate.

## Discussion

In this review, we collectively searched publications related to the effect of glucosamine on knee OA written not only in English but also in Japanese and other Asian languages, resulting in 18 collected articles. Previously, a review by Cochrane on glucosamine therapy for OA reported 25 articles in 2008, which covered the 1966–2008 period [8]. In our meta-analysis, we selected studies after 2003 to identify more recent findings on glucosamine effects. Our meta-analysis revealed favorable results for glucosamine compared to placebos, especially in terms of VAS pain score and JKOM. Because several reviews concluded that the effect of glucosamine varied and was not definite, our results provide novel information about glucosamine [13, 14]. At the same time, note that the collective effect of glucosamine is small.

As reported in previous systematic reviews, our series also involves various types of glucosamine products. Although the dose of glucosamine was 1500 mg/day in most of the studies, the biological activities of ingested glucosamine were not necessarily equivalent. In particular, better results obtained from Rottaham/Madacus products depend on their crystalline form, which is more suitable for absorption. Our meta-analysis for pain VAS did not include studies using Rottaham/Madacus product and still showed significant effect of glucosamine on pain symptom. Therefore, in spite of large variations in its product, we assume that glucosamine in general has a favorable effect on pain symptom. Consistent with our results, Towheed reported statistically significant effects of glucosamine on pain, when they pooled various pain measurement methods (SMD = − 0.47; 95% CI = − 0.72 to − 0.23), while the effect was not significant for pain measured by WOMAC pain sub-score (SMD = − 0.06; 95% CI = − 0.14 to 0.03) [8]. Given that there is a tendency toward consistency of results in recent studies, the findings of our current meta-analysis should be robust.

In our systematic review of the effect of glucosamine on health-related QOL among patients with knee OA, we gathered information about the effect of glucosamine, as measured by WOMAC and JKOM. The JKOM was developed based on the WOMAC and SF-36 to reflect pain, mobility, and participation of patients with knee OA [38]. It includes 25 questions divided into 4 categories: pain and stiffness in knees, condition in daily life, general activities, and health conditions. Our meta-analysis showed a non-significant effect on WOMAC scores but a significant effect on JKOM scores in favor of glucosamine. While we should be cautious that the study size was relatively small (total of 50 subjects from 3 trials), it is possible that JKOM is more sensitive than WOMAC for measuring QOL of patients with knee OA. In the process of designing JKOM, the chance of confounding effect among questionnaire items was minimized, so that the sensitivity of the scale is secured for various levels of severity of knee OA [39]. Further studies using JKOM to assess the effect of glucosamine may provide more robust results about the efficacy of glucosamine.

While we confirmed the effect of glucosamine, the placebo group also showed improvement in outcome scales during the trial in most studies. This could be either due to the natural course of knee OA, placebo effects, or effects of other treatments the subjects chose, some of which researchers may have not been aware during the trial. These trends were commonly observed in other studies on musculoskeletal organs. We should be aware of this consideration when evaluating the results of the effect of any drug on knee OA symptoms.

Since knee OA is a slowly progressive pathology, the duration of intervention is critical in the assessment of efficacy. Among the articles reviewed, most articles have extended follow-up of more than 12 weeks. This seems to be an appropriate duration to assess the effects on the symptoms and performance of the knee. However, from the viewpoint of assessing cartilage preservation, 12 weeks is too short to evaluate results. Therefore, some studies took more than 2 years for evaluation, while some others utilized biochemical markers in the joint fluid to estimate the effect. The severity of OA at the initial time point is crucial to evaluate long-term structural changes in joint cartilage. While the LEG study did not show a joint space preservation effect in a mild OA population [31], Raynauld et al. recently reported positive effects among OA knees where medial meniscal extrusion exists at baseline [40]. Given the paucity of studies dealing with cartilage preservation, we could not perform a meta-analysis this time. Because long-term intervention by supplements with sufficient compliance is difficult in terms of compliance, we need a new method to evaluate the condition of cartilage within a short time period, such as evaluation by magnetic resonance imaging or using reliable fluid biomarkers [41, 42].

The current systematic review had several limitations. The products used in half of our series (nine studies) contain bioactive supplement other than glucosamine, which makes it difficult to assess the glucosamine effect alone. In particular, because chondroitin sulfate, involved in six studies, is expected to provide anti-inflammatory and joint preserving effects, the results from the combination recipe may involve a synergistic effect of glucosamine and chondroitin. Moreover, we found that the combination recipe tends have a larger effect on pain compared with glucosamine alone. Further study may be needed to explore an ideal combination with glucosamine. We tried to collect non-English articles but could only obtain Japanese and Chinese papers. Considering that glucosamine is now a supplement used globally, more studies might have been conducted in other countries. In addition, we had difficulty in selecting the outcomes for our meta-analysis. Because of the variation in the outcome scales used in each study, we could only conduct a meta-analysis for a very limited part of the results.

In summary, we reviewed recent RCT studies to examine the effect of glucosamine and found favorable effects of the supplement for pain alleviation with limited effect size. Our results suggest that clinicians should consider glucosamine as a supplement for patients with OA. Optimized tools for measurement are required to evaluate the effect of glucosamine on the activities of daily living of subjects. Long-term administration and observation using consistent scales and measurement of cartilage preservation are suggested for future studies.

## References

[1] Lawrence RC, Helmick CG, Arnett FC, Deyo RA, Felson DT, Giannini EH, Heyse SP, Hirsch R, Hochberg MC, Hunder GG, Liang MH, Pillemer SR, Steen VD, Wolfe F (1998). Estimates of the prevalence of arthritis and selected musculoskeletal disorders in the United States. Arthritis Rheum.

[2] Yoshimura N, Muraki S, Oka H, Mabuchi A, En-Yo Y, Yoshida M, Saika A, Yoshida H, Suzuki T, Yamamoto S, Ishibashi H, Kawaguchi H, Nakamura K, Akune T (2009). Prevalence of knee osteoarthritis, lumbar spondylosis, and osteoporosis in Japanese men and women: the research on osteoarthritis/osteoporosis against disability study. J Bone Miner Metab.

[3] Muraki S, Akune T, Oka H, En-yo Y, Yoshida M, Saika A, Suzuki T, Yoshida H, Ishibashi H, Tokimura F, Yamamoto S, Nakamura K, Kawaguchi H, Yoshimura N (2010). Association of radiographic and symptomatic knee osteoarthritis with health-related quality of life in a population-based cohort study in Japan: the ROAD study. Osteoarthr Cartil.

[4] Wang SY, Olson-Kellogg B, Shamliyan TA, Choi JY, Ramakrishnan R, Kane RL (2012). Physical therapy interventions for knee pain secondary to osteoarthritis: a systematic review. Ann Intern Med.

[5] McAlindon TE, Bannuru RR, Sullivan MC, Arden NK, Berenbaum F, Bierma-Zeinstra SM, Hawker GA, Henrotin Y, Hunter DJ, Kawaguchi H, Kwoh K, Lohmander S, Rannou F, Roos EM, Underwood M (2014). OARSI guidelines for the non-surgical management of knee osteoarthritis. Osteoarthr Cartil.

[6] Jevsevar DS (2013). Treatment of osteoarthritis of the knee: evidence-based guideline, 2nd edition. J Am Acad Orthop Surg.

[7] Molloy IB, Martin BI, Moschetti WE, Jevsevar DS (2017). Effects of the length of stay on the cost of total knee and total hip arthroplasty from 2002 to 2013. J Bone Joint Surg Am.

[8] Towheed TE, Maxwell L, Anastassiades TP, Shea B, Houpt J, Robinson V, Hochberg MC, Wells G (2005). Glucosamine therapy for treating osteoarthritis. Cochrane Database Syst Rev.

[9] Wandel S, Juni P, Tendal B, Nuesch E, Villiger PM, Welton NJ, Reichenbach S, Trelle S (2010). Effects of glucosamine, chondroitin, or placebo in patients with osteoarthritis of hip or knee: network meta-analysis. BMJ.

[10] McAlindon TE, LaValley MP, Gulin JP, Felson DT (2000). Glucosamine and chondroitin for treatment of osteoarthritis: a systematic quality assessment and meta-analysis. JAMA.

[11] Snijders GF, den Broeder AA, van Riel PL, Straten VH, de Man FH, van den Hoogen FH, van den Ende CH, Group NS (2011) Evidence-based tailored conservative treatment of knee and hip osteoarthritis: between knowing and doing. Scand J Rheumatol 40 (3):225–231. doi:10.3109/03009742.2010.530611 10.3109/03009742.2010.530611 21261551

[12] Gallagher B, Tjoumakaris FP, Harwood MI, Good RP, Ciccotti MG, Freedman KB (2015). Chondroprotection and the prevention of osteoarthritis progression of the knee: a systematic review of treatment agents. Am J Sports Med.

[13] Vangsness CT, Spiker W, Erickson J (2009). A review of evidence-based medicine for glucosamine and chondroitin sulfate use in knee osteoarthritis. Arthroscopy.

[14] Eriksen P, Bartels EM, Altman RD, Bliddal H, Juhl C, Christensen R (2014). Risk of bias and brand explain the observed inconsistency in trials on glucosamine for symptomatic relief of osteoarthritis: a meta-analysis of placebo-controlled trials. Arthritis Care Res (Hoboken).

[15] Bruyere O, Cooper C, Pelletier JP, Maheu E, Rannou F, Branco J, Luisa Brandi M, Kanis JA, Altman RD, Hochberg MC, Martel-Pelletier J, Reginster JY (2016) A consensus statement on the European Society for Clinical and Economic Aspects of Osteoporosis and Osteoarthritis (ESCEO) algorithm for the management of knee osteoarthritis—from evidence-based medicine to the real-life setting. Semin Arthritis Rheum 45(4 Suppl):S3–S11. 10.1016/j.semarthrit.2015.11.010 10.1016/j.semarthrit.2015.11.010 26806188

[16] Hayami Y, Shichikawa K (2003). Effect of glucosamine, shark cartilage extract, and roots extract on knee osteoarthritis. Rinsho-to-Kenkyu.

[17] Usha PR, Naidu MU (2004). Randomised, double-blind, parallel, placebo-controlled study of oral glucosamine, methylsulfonylmethane and their combination in osteoarthritis. Clin Drug Investig.

[18] Cibere J, Kopec JA, Thorne A, Singer J, Canvin J, Robinson DB, Pope J, Hong P, Grant E, Esdaile JM (2004). Randomized, double-blind, placebo-controlled glucosamine discontinuation trial in knee osteoarthritis. Arthritis Rheum.

[19] McAlindon T, Formica M, LaValley M, Lehmer M, Kabbara K (2004). Effectiveness of glucosamine for symptoms of knee osteoarthritis: results from an internet-based randomized double-blind controlled trial. Am J Med.

[20] Kajimoto O, Miyabayashi N, Nakagawa S, Kajimoto Y (2005). A efficacy of tablet containing glucosamine hydrochloride on knee osteoarthritis. J New Rem & Clin.

[21] Clegg DO, Reda DJ, Harris CL, Klein MA, O'Dell JR, Hooper MM, Bradley JD, Bingham CO, Weisman MH, Jackson CG, Lane NE, Cush JJ, Moreland LW, Schumacher HR, Oddis CV, Wolfe F, Molitor JA, Yocum DE, Schnitzer TJ, Furst DE, Sawitzke AD, Shi H, Brandt KD, Moskowitz RW, Williams HJ (2006). Glucosamine, chondroitin sulfate, and the two in combination for painful knee osteoarthritis. N Engl J Med.

[22] Hatano K, Hayashida K, Nakagawa S, Miyakuni Y (2006). Effects and safety of soymilk beverage containing N-acetyl glucosamine on osteoarthritis. Jpn Pharmacol Ther.

[23] Herrero-Beaumont G, Ivorra JA, Del Carmen Trabado M, Blanco FJ, Benito P, Martin-Mola E, Paulino J, Marenco JL, Porto A, Laffon A, Araujo D, Figueroa M, Branco J (2007). Glucosamine sulfate in the treatment of knee osteoarthritis symptoms: a randomized, double-blind, placebo-controlled study using acetaminophen as a side comparator. Arthritis Rheum.

[24] Frestedt JL, Walsh M, Kuskowski MA, Zenk JL (2008). A natural mineral supplement provides relief from knee osteoarthritis symptoms: a randomized controlled pilot trial. Nutr J.

[25] Inuzuka M, Hosono N, Ninomiya C, Itanami E, Sugano T (2010). The clinical safety and efficacy of oral MSM tablet administration was evaluated by the randomized controlled trial against arthralgia. Clin Pharmacol Therapy.

[26] Nakasone Y, Watabe K, Watanabe K, Tomonaga A, Nagaoka I, Yamamoto T, Yamaguchi H (2011). Effect of a glucosamine-based combination supplement containing chondroitin sulfate and antioxidant micronutrients in subjects with symptomatic knee osteoarthritis: a pilot study. Exp Ther Med.

[27] Kanzaki N, Saito K, Maeda A, Kitagawa Y, Kiso Y, Watanabe K, Tomonaga A, Nagaoka I, Yamaguchi H (2012). Effect of a dietary supplement containing glucosamine hydrochloride, chondroitin sulfate and quercetin glycosides on symptomatic knee osteoarthritis: a randomized, double-blind, placebo-controlled study. J Sci Food Agric.

[28] Gokan N, Suzuki N, Shiizuka K, Yamamoto K, Takara T (2011). The effect of the dietary supplement containing with both glucosamine and chondroitin sulfate on knee joint pain. J New Rem Clin.

[29] Nieman DC, Shanely RA, Luo B, Dew D, Meaney MP, Sha W (2013). A commercialized dietary supplement alleviates joint pain in community adults: a double-blind, placebo-controlled community trial. Nutr J.

[30] Yokoi K, Fujimoto Y (2013) [Effect of the dietary containing N-acetyl glucosamine on knee pain and cartilage matabolism]. J New Rem Clin 62 (9):256–266

[31] Fransen M, Agaliotis M, Nairn L, Votrubec M, Bridgett L, Su S, Jan S, March L, Edmonds J, Norton R, Woodward M, Day R, group Lsc (2015). Glucosamine and chondroitin for knee osteoarthritis: a double-blind randomised placebo-controlled clinical trial evaluating single and combination regimens. Ann Rheum Dis.

[32] Tsuji T, Yoon J, Kitano N, Okura T, Tanaka K (2016). Effects of N-acetyl glucosamine and chondroitin sulfate supplementation on knee pain and self-reported knee function in middle-aged and older Japanese adults: a randomized, double-blind, placebo-controlled trial. Aging Clin Exp Res.

[33] Sterzi S, Giordani L, Morrone M, Lena E, Magrone G, Scarpini C, Milighetti S, Pellicciari L, Bravi M, Panni I, Ljoka C, Bressi F, Foti C (2016). The efficacy and safety of a combination of glucosamine hydrochloride, chondroitin sulfate and bio-curcumin with exercise in the treatment of knee osteoarthritis: a randomized, double-blind, placebo-controlled study. Eur J Phys Rehabil Med.

[34] Qiu G, Weng X, Zhang K, Zhou Y, Lou S, Wang Y, Li W, Zhang H, Liu Y (2005). A multi-central, randomized, controlled clinical trial of glucosamine hydrochloride/sulfate in the treatment of knee osteoarthritis. Natl Med J China.

[35] Bruyere O, Pavelka K, Rovati LC, Deroisy R, Olejarova M, Gatterova J, Giacovelli G, Reginster JY (2004). Glucosamine sulfate reduces osteoarthritis progression in postmenopausal women with knee osteoarthritis: evidence from two 3-year studies. Menopause.

[36] Bruyere O, Pavelka K, Rovati LC, Gatterova J, Giacovelli G, Olejarova M, Deroisy R, Reginster JY (2008). Total joint replacement after glucosamine sulphate treatment in knee osteoarthritis: results of a mean 8-year observation of patients from two previous 3-year, randomised, placebo-controlled trials. Osteoarthr Cartil.

[37] Sawitzke AD, Shi H, Finco MF, Dunlop DD, Harris CL, Singer NG, Bradley JD, Silver D, Jackson CG, Lane NE, Oddis CV, Wolfe F, Lisse J, Furst DE, Bingham CO, Reda DJ, Moskowitz RW, Williams HJ, Clegg DO (2010). Clinical efficacy and safety of glucosamine, chondroitin sulphate, their combination, celecoxib or placebo taken to treat osteoarthritis of the knee: 2-year results from GAIT. Ann Rheum Dis.

[38] Akai M, Doi T, Fujino K, Iwaya T, Kurosawa H, Nasu T (2005). An outcome measure for Japanese people with knee osteoarthritis. J Rheumatol.

[39] Doi T, Akai M, Fujino K, Iwaya T, Kurosawa H, Hayashi K, Marui E (2008). Effect of home exercise of quadriceps on knee osteoarthritis compared with nonsteroidal antiinflammatory drugs: a randomized controlled trial. Am J Phys Med Rehabil.

[40] Raynauld JP, Pelletier JP, Abram F, Dodin P, Delorme P, Martel-Pelletier J (2016). Long-term effects of glucosamine and chondroitin sulfate on the progression of structural changes in knee osteoarthritis: six-year follow-up data from the osteoarthritis initiative. Arthritis Care Res (Hoboken).

[41] Eckstein F, Guermazi A, Gold G, Duryea J, Hellio Le Graverand MP, Wirth W, Miller CG (2014). Imaging of cartilage and bone: promises and pitfalls in clinical trials of osteoarthritis. Osteoarthr Cartil.

[42] Mobasheri A, Bay-Jensen AC, van Spil WE, Larkin J, Levesque MC (2017). Osteoarthritis year in review 2016: biomarkers (biochemical markers). Osteoarthr Cartil.

